# EST analysis reveals putative genes involved in glycyrrhizin biosynthesis

**DOI:** 10.1186/1471-2164-11-268

**Published:** 2010-04-28

**Authors:** Ying Li, Hong-Mei Luo, Chao Sun, Jing-Yuan Song, Yong-Zhen Sun, Qiong Wu, Ning Wang, Hui Yao, André Steinmetz, Shi-Lin Chen

**Affiliations:** 1 Institute of Medicinal Plant Development (IMPLAD), Chinese Academy of Medical Sciences & Peking Union Medical College, No.151, Malianwa North Road, HaiDian District, Beijing 100193, China; 2 Hubei College of Traditional Chinese Medicine, No. 1, Huangjia Lake West Road, Hongshan District, Wuhan City, Hubei Province 430065, China; 3 Centre de Recherche Public-Santé, Luxembourg, L-1526 Luxembourg

## Abstract

**Background:**

*Glycyrrhiza uralensis *is one of the most popular medicinal plants in the world and is also widely used in the flavoring of food and tobacco. Due to limited genomic and transcriptomic data, the biosynthetic pathway of glycyrrhizin, the major bioactive compound in *G. uralensis*, is currently unclear. Identification of candidate genes involved in the glycyrrhizin biosynthetic pathway will significantly contribute to the understanding of the biosynthetic and medicinal chemistry of this compound.

**Results:**

We used the 454 GS FLX platform and Titanium regents to produce a substantial expressed sequence tag (EST) dataset from the vegetative organs of *G. uralensis*. A total of 59,219 ESTs with an average read length of 409 bp were generated. 454 ESTs were combined with the 50,666 *G. uralensis *ESTs in GenBank. The combined ESTs were assembled into 27,229 unique sequences (11,694 contigs and 15,535 singletons). A total of 20,437 unique gene elements representing approximately 10,000 independent transcripts were annotated using BLAST searches (e-value ≤ 1e-5) against the SwissProt, KEGG, TAIR, Nr and Nt databases. The assembled sequences were annotated with gene names and Gene Ontology (GO) terms. With respect to the genes related to glycyrrhizin metabolism, genes encoding 16 enzymes of the 18 total steps of the glycyrrhizin skeleton synthesis pathway were found. To identify novel genes that encode cytochrome P450 enzymes and glycosyltransferases, which are related to glycyrrhizin metabolism, a total of 125 and 172 unigenes were found to be homologous to cytochrome P450s and glycosyltransferases, respectively. The cytochrome P450 candidate genes were classified into 32 CYP families, while the glycosyltransferase candidate genes were classified into 45 categories by GO analysis.  Finally, 3 cytochrome P450 enzymes and 6 glycosyltransferases were selected as the candidates most likely to be involved in glycyrrhizin biosynthesis through an organ-specific expression pattern analysis based on real-time PCR.

**Conclusions:**

Using the 454 GS FLX platform and Titanium reagents, our study provides a high-quality EST database for *G. uralensis*. Based on the EST analysis, novel candidate genes related to the secondary metabolite pathway of glycyrrhizin, including novel genes encoding cytochrome P450s and glycosyltransferases, were found. With the assistance of organ-specific expression pattern analysis, 3 unigenes encoding cytochrome P450s and 6 unigenes encoding glycosyltransferases were selected as the candidates most likely to be involved in glycyrrhizin biosynthesis.

## Background

The sequencing and analysis of expressed sequence tags (ESTs) has been a primary tool for the discovery of novel genes in plants, especially in non-model plants for which full genome sequences are not currently available [1]. EST sequencing represents a rapid and cost-effective method for analyzing the transcribed regions of genomes. EST analysis is also a powerful tool for the discovery of genes involved in plant secondary metabolism. The 454 GS FLX sequencing technology has made EST-based resources more readily accessible for non-model organism transcriptomes [2,3]. Our experimental focus for this study was *Glycyrrhiza uralensis *Fisch. ex DC., which is one of the most ancient medicinal herbs and has been used as a Chinese herbal medicine to treat infectious diseases for over 3,000 years [4]. This herb has been extensively studied and is widely used as a flavoring agent, medicament and tobacco additive. Many of the biological activities of the bioactive constituents of *G. uralensis *have been investigated, including the protection against hepatotoxicity [5,6], anti-ulcer effects [7], anti-inflammatory [8] and anti-tumor promoting activities [9]. This herb also exhibits antiviral activity against various DNA and RNA viruses, including herpes simplex virus [10], HIV [11,12] and severe acute respiratory syndrome (SARS)-associated coronavirus [13].

These biological activities of *G. uralensis *have been primarily attributed to two of its components, flavonoids and saponins. Our research interests primarily concern glycyrrhizin, an oleanane-type triterpene saponin and a well-known natural sweetener that is fifty times sweeter than sugar [14]. Although the various chemical and pharmacological properties of glycyrrhizin in *G. uralensis *have been extensively studied, the biosynthetic pathway of this compound remains poorly understood. Two functional genes encoding squalene synthase (SQS) have been isolated from *G. uralensis *[15]. Two cytochrome P450 genes have also been isolated from *G. uralensis *based on the traditional EST sequencing method [16]. *CYP88D6*, a cytochrome P450 monooxygenase, was characterized by *in vitro *enzymatic activity assays and was shown to catalyze the oxidation of β-amyrin at C-11 to produce 11-oxo-β-amyrin, a possible biosynthetic intermediate in the glycyrrhizin biosynthetic pathway [16]. Another cytochrome P450 from *G. uralensis*, *CYP93E3*, possesses β-amyrin 24-hydroxylase activity in *in vitro *enzymatic activity assays [16]. A functional β-amyrin synthase gene (bAS) has been isolated from *G. glabra *[17,18]. Thus far, only one glycosyltransferase in the *Glycyrrhiza *genus, the isoflavonoid glucosyltransferase in *G. echinata*, has been identified [19]. However, no progress has been made in the identification of the genes involved in the glycosylation of glycyrrhetinic acid to produce glycyrrhizin. Transcriptome sequencing would provide a foundation for detailed studies of gene expression and genetic connectivity with respect to plant secondary metabolism.

In our study, we constructed a cDNA library using the vegetative organs of five-year-old wild *G. uralensis *cultivated from the city of Yanchi in the Ningxia province of China, one of the most famous areas for the production of wild *G. uralensis*. The library was sequenced using the 454 GS FLX platform and Titanium reagents. There are currently 50,666 *G. uralensis *ESTs in the GenBank (GuEST) dbEST database, which were determined using conventional sequencing techniques [20]. In our study, we increased this collection with an additional 59,219 ESTs generated from 454 GS FLX Titanium sequencing. Bioinformatic analyses indicated that almost all of the genes involved in the biosynthesis of the glycyrrhizin skeleton were within the combined EST database, except for mevalonate kinase (EC 2.7.1.36) and DXP synthase (EC 2.2.1.7). Additionally, a pool of candidate genes for cytochrome P450s and glycosyltransferases was established, containing 125 and 172 unigenes, respectively. Finally, using an organ-specific expression pattern analysis, a few unigenes were selected as the candidates most likely to be responsible for glycyrrhizin skeleton modifications. The method described here is a cost-effective technology for the identification of novel genes in non-model organisms that serve as medicinal plants. Furthermore, the ESTs and unigenes described in our study constitute an important resource for future studies of the molecular genetics and functional genomics of *G. uralensis*.

## Results and Discussion

### 454 sequencing and assembly

**Figure 1:**
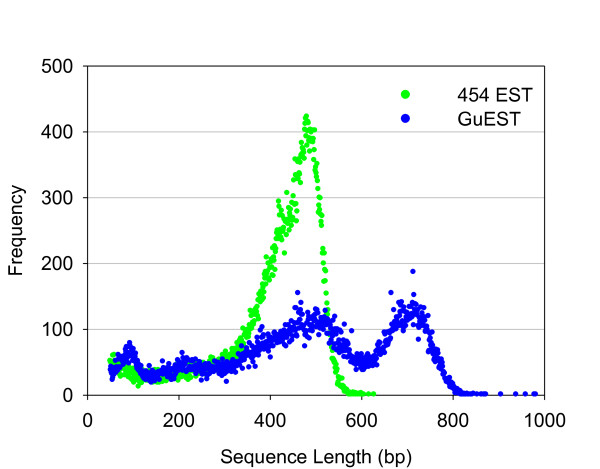
**Sequence length distribution of *G. uralensis *454 ESTs and GenBank ESTs**.

**Figure 2:**
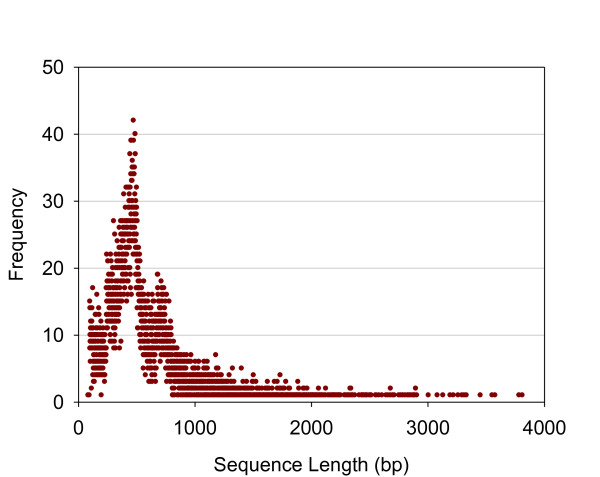
**Contig length distribution**.

**Figure 3:**
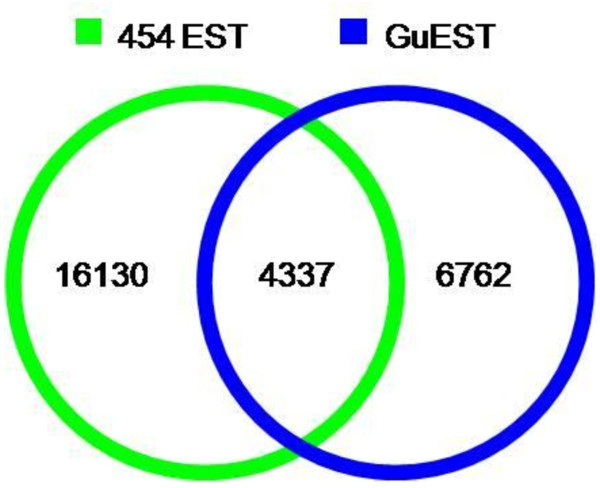
**Comparison of the 454 ESTs and GuESTs**. The overlapping section corresponds to unigenes composed of ESTs in the two datasets.

A *G. uralensis *cDNA library was constructed from a pool of mRNA isolated from the vegetative organs of *G. uralensis *and was sequenced using the 454 GS FLX platform and Titanium regents. After the initial quality filtering, this one-eighth sequencing run produced 59,219 high-quality reads (HQ reads) with an average length of 409 bp (mode, 498; range, 10-653) and a total length of 24.2 Mb. Of these HQ reads, 57,997 exceeded our minimal quality standards (SMART primer filtering; length threshold of 50 bp) and were used in the assembly. The large portion of reads used after trimming and filtering indicates that most of the sequences were used in the assembly and that the quality of the data was considerably high. An additional 50,666 *G. uralensis *ESTs were downloaded from GenBank (GuEST) and combined with our 454 derived ESTs. A summary of these two EST datasets is given in Table 1. Figure 1 illustrates the sequence length distributions of the ESTs derived from 454 sequencing and GenBank. Assembly of the trimmed, size-selected ESTs produced 11,694 contigs with a mean length of 571 bp and a range of 85 - 3,812 bp, as well as an additional 15,535 singletons, for a total of 27,229 unigenes (contigs and singletons) (Table 2). The length distribution of the contigs is shown in Figure 2. The assembly produced a substantial number of large contigs (10,868 contigs were > 200 bp in length). Approximately 16% of the unigenes (4,337 out of 27,229) were composed of the 454 ESTs and GuESTs data (Figure 3). The 454 sequencing identified 16,130 novel unigenes, bringing the total number of different *G. uralensis *unigenes in the databases to 27,229. This number should cover the vast majority of genes from this species, including those expressed at low levels.

**Table 1:** Summary of *G. uralensis *ESTs derived from 454 sequencing and GenBank

	454 EST	GuEST
HQ reads^a^	59,219	50,666
total bases of HQ reads^a^	24,231,155 bp	24,211,504 bp
average HQ read length^a^	409 ± 120 bp	478 ± 198 bp
average quality	32	
reads used in assembly (after trimming and filtering)	57,997	50,451
bases used in assembly	23,526,020 bp	24,099,889 bp
	(97.1%)	(99.5%)

^a^HQ indicates high-quality

**Table 2:** Summary of G. uralensis EST assembly

Contigs	
reads assembled as contigs	92,221
bases assembled as contigs	39,171,687 bp
number of contigs	11,694
total length of contigs	6,673,089 bp
average length of contigs	571 ± 368 bp
range of contig lengths	85 - 3,812 bp
contigs above 200 bp	10,868
N50 contig size	816 bp
depth of contigs	5.84
**Singletons**	
number of singletons	15,535
total length of singletons	5,687,305 bp
average length of singletons	366 ± 163 bp
range of singleton lengths	50 - 853 bp
singletons above 200 bp	12,290

**Unigenes**^a^	
number of unigenes	27,229
total length of unigenes	12,360,394 bp
unigenes above 200 bp	23,158

^a^unigenes includes contigs and singletons

For homology searches against known genes, unigenes longer than 200 bp are widely accepted as valid sources, making them sufficient for the effective assignment of functional annotations [21]. Most of the unigene sequences were longer than 200 bp and were valid in this study (85%; 23,158/27,229).

### Functional annotation

Our annotation method was based on sequence homology searches and the annotations that accompanied them. Its aim was to capture the most informative and complete annotation possible. Additional file 1 shows the hit numbers and percentages relative to those of the major public databases. These annotation statistics show all the unigenes annotated by the BLAST [22] search against the public protein and nucleotide databases (SwissProt [23], KEGG [24], TAIR [25], Nr [26] and Nt [27]) where the e-value threshold was set at 1e-5. Of the 27,229 unigenes, 20,437 unigenes had at least one hit within these databases. The remaining unigenes (24.9%) that were not annotated likely comprise *G. uralensis*-specific genes, as well as genes with homologs in other species whose corresponding biological functions have not yet been investigated.

### Gene Ontology classification

**Figure 4:**
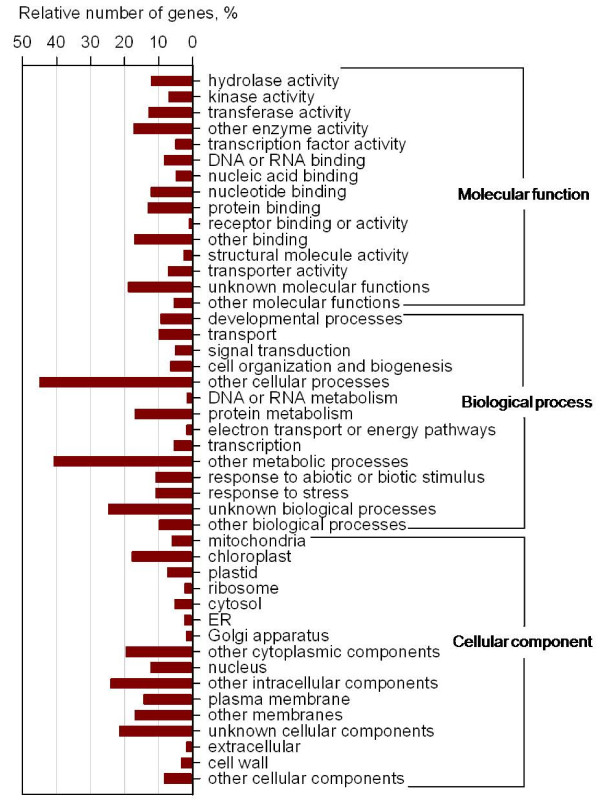
**Functional annotation of the unigenes based on GO categories**.

As a result of the completed genomic sequencing of the plant *Arabidopsis thaliana*, the currently available expressed sequences have been invaluable in defining the correct components of the gene structure in this species [28]. To obtain an overview of the gene functions of the ESTs from *G. uralensis*, the annotated unigenes were categorized according to Gene Ontology (GO) on the basis of the TAIR GO slim provided by TAIR [29]. The *G. uralensis *unigenes were compared with the *Arabidopsis *proteome [25] using BlastX [22]. We used the annotations of each unigene to assign it to the GO categories for Molecular Function, Biological Process and Cellular Component (Figure 4). The GO analysis showed that the functions of the identified genes are involved various biological processes. A large number of hydrolases, kinases and transferases were annotated, which suggests that our study may allow for the identification of novel genes involved in the secondary metabolite synthesis pathways.

### Putative genes related to the biosynthesis of glycyrrhizin

In this study, our primary goal was to identify genes involved in the glycyrrhizin biosynthetic pathway (Additional file 2 and Table 3) [16,30]. The biosynthesis of glycyrrhizin involves the synthesis of dimethylallyl diphosphate (DMAPP) and isopentenyl diphosphate (IPP), the biochemically active isoprene units of all terpenoids [30]. This step is followed by the synthesis of the triterpene skeleton, also known as β-amyrin [30], and then by a series of oxidative reactions and glucuronylations, which produce glycyrrhizin. The precise order of the intermediate products is still unknown [16].

**Table 3:** Number of putative unigenes and ESTs involved in glycyrrhizin skeleton biosynthesis^a^

EC^b^	Enzyme name	Number of Unigenes	Number of ESTs	Number of 454 ESTs	Number of GuESTs
2.3.1.9	acetyl-CoA acetyltransferase	3	30	28	2
2.3.3.10	HMG-CoA synthase	2	11	5	6
1.1.1.34	HMG-CoA reductase	2	17	16	1
2.7.1.36	mevalonate kinase	0	0	0	0
2.7.4.2	phosphomevalonate kinase	2	5	4	1
4.1.1.33	mevalonate-5-diphosphate decarboxylase	1	1	1	0
2.2.1.7	DXP synthase	0	0	0	0
1.1.1.267	DXP reductoisomerase	3	6	4	2
2.7.7.60	MEP cytidylyltransferase	2	2	2	0
2.7.1.148	CDP-ME kinase	1	1	1	0
4.6.1.12	MECDP synthase	1	6	4	2
1.17.7.1	4-hydroxy-3-methylbut-2-enyl-diphosphate synthase	2	25	25	0
1.17.1.2	4-hydroxy-3-methylbut-2-enyl-diphosphate reductase	1	24	20	4
5.3.3.2	isopentenyl-PP isomerase	1	63	31	32
2.5.1.10	farnesyl diphosphate synthase	1	1	1	0
2.5.1.21	squalene synthase	1	1	1	0
1.14.99.7	squalene monooxygenase	4	23	23	0
5.4.99.-	β-amyrin synthase	1	1	1	0

^a^BLAST against the SwissProt and KEGG databases; ^b^Enzyme code

In the early stage of active isoprene unit formation, plants have the ability to produce DMAPP and IPP using two pathways, the mevalonate pathway (MVA pathway) and the methylerythritol phosphate pathway (MEP pathway). In plants, these two pathways appear to be separate; enzymes of the MVA pathway are found in the cytosol, whereas enzymes of the MEP pathway are localized in plastids. Triterpenoids are known to be formed by the MVA pathway because they are cytosolic products. However, there are examples where the two pathways can act cooperatively to create a molecule [30]. No progress has been made toward determining the precise source of isoprene units in glycyrrhizin biosynthesis. Using a BLAST [22] search against the SwissProt [23] and KEGG [24] databases, we found the genes encoding all of the enzymes from both of these two pathways in the EST database, except for mevalonate kinase (EC 2.7.1.36), which is located in the MVA pathway, and DXP synthase (EC 2.2.1.7), which is located in the MEP pathway. In this study, we found all of the putative genes encoding the enzymes involved in the triterpene skeleton β-amyrin synthesis step: farnesyl diphosphate synthase (FPS), squalene synthase (SQS), squalene monooxygenase and β-amyrin synthase (bAS). The enzymes involved in the biosyntheses of the isoprene unit and the triterpene skeleton are listed in Table 3. A list of putative unigenes involved in the glycyrrhizin biosynthetic pathway is shown in Additional file 3.

### Cytochrome P450 and glycosyltransferase

Glycyrrhizin is derived from the triterpene β-amyrin, which is an initial from product of thethe cyclization of 2, 3-oxidosqualene. The subsequent steps in glycyrrhizin biosynthesis include a series of oxidative and glycosyl transfer reactions. We have little knowledge of the later steps in the glycyrrhizin biosynthetic pathway, which include multiple oxidation and glycosylation steps that are catalyzed by enzymes from the cytochrome P450 (CYP) and glycosyltransferase superfamilies, respectively.

**Figure 5:**
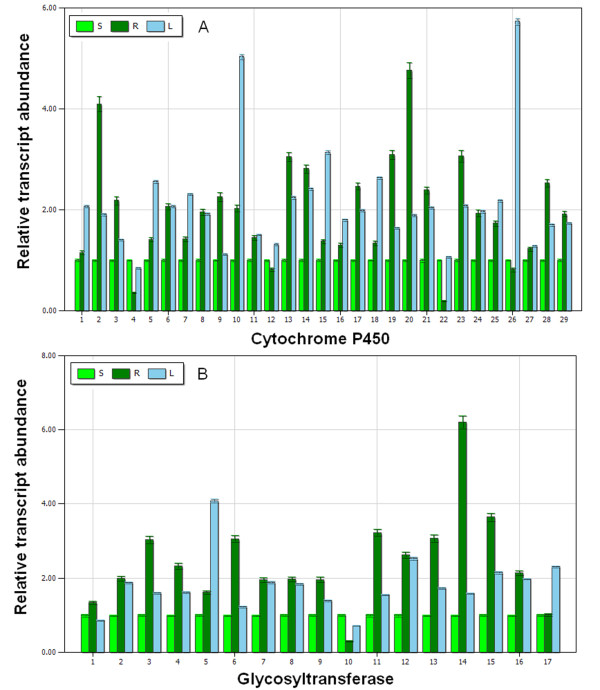
**Real-time PCR analysis of cytochrome P450s (A) and glycosyltransferases (B) in different plant organs**. R represents the root, S represents the stem, and L represents the leaf. The corresponding cytochrome P450 and glycosyltransferases contigs represented by the numbers are listed in Additional file 5. A) The gene expression of *cytochrome P450*s in different organs. B) The gene expression of *glycosyltransferases *in different organs.

Cytochrome P450 is a very large and diverse superfamily of hemoproteins that are found in all higher organisms [31,32]. Plant P450s catalyze many different reactions involved in the biosynthesis of secondary metabolites, including terpenoids [33]. Some members of the CYP88 and CYP93 families have been shown to act on β-amyrin or related triterpene substrates with unique reaction specificities [16,34-36]. Thus far, all known cytochrome P450s that act on triterpenes and sterols have been classified into two clans: the CYP71 clan and the CYP85 clan, which includes CYP93 and CYP88, respectively [16,34,37-39]. Only two *CYP *genes of *G. uralensis *have been identified [16]. An organ-specific transcript profiling approach was used in other studies to identify *CYP88D6*, which catalyzes the oxidation of β-amyrin at C-11 to produce 11-oxo-β-amyrin in the glycyrrhizin biosynthetic pathway. The expression profile of *CYP88D6 *was consistent with the organ-specific accumulation pattern of glycyrrhizin [16]; a higher level of expression was seen in the root than in the stem and leaf. By mining the EST database, we found 125 unigenes (500 ESTs) annotated as putative *CYP *genes, which were further classified into 32 CYP families and 47 subfamilies (Additional file 4). To narrow down the candidate cytochrome P450s, these unigenes were further screened according to their classification. In the candidate P450 dataset, two unigenes (23 ESTs) were annotated as CYP88, while six unigenes (30 ESTs) were annotated as CYP93. In total, 29 contigs annotated as cytochrome P450 and belonging to the CYP71 and CYP85 clans (Additional file 5), were chosen for organ-specific expression pattern assays (Figure 5A). Contig01314 (No. 2 in Figure 5A) was exactly the same as the *CYP88D6 *gene according to the BLAST annotation results. An additional 3 unigenes had a similar organ-specific expression pattern as *CYP88D6*, including contig06734 (No. 19 in Figure 5A), contig07137 (No. 20 in Figure 5A) and contig07899 (No. 23 in Figure 5A). However, additional experiments are needed to determine which of these unigenes participate in glycyrrhizin biosynthesis.

Glycosyltransferases, a ubiquitous family of enzymes, catalyze reactions involving the transfer of a nucleotide-activated sugar moiety onto another molecule [40]. These enzymes are encoded by a large multigene family; approximately 120 secondary metabolism glycosyltransferase genes have been identified in *Arabidopsis*. The conjugation of a sugar moiety to a substrate is called glycosylation, which is a process that contributes to the synthesis of glucidic polymers, glycoproteins and glycolipids. Glycosyltransferases often use specific substrates in the glycosylation reaction and are relevant for the synthesis of secondary metabolites. No genes encoding relevant glycosyltransferases have been identified in *Glycyrrhiza*.

Using BLAST searches, approximately 172 unigenes (1205 ESTs) in our study showed sequence similarities to glycosyltransferase (EC: 2.4.-.-) in the KEGG database. According to the GO category analysis, these unigenes were classified into 45 categories (Additional file 6). Among these categories, 27 unigenes (83 ESTs) encoded for UDP-glucosyltransferases, which is obviously involved in the biosynthesis of secondary metabolites. We also pick up unigenes annotated as glucuronosyltransferases because glycyrrhizin is composed of aglycone glycyrrhetic acid and two glucuronic acid units. We found 11 unigenes (33 ESTs) that encoded glucuronosyltransferases, and it is possible that these are involved in the last steps of glycyrrhizin biosynthesis. From these two categories, 17 contigs were chosen (Additional file 5) for organ-specific expression pattern analysis by real-time PCR (Figure 5B). The expression patterns of 6 glycosyltransferase unigenes were similar to that of *CYP88D6*. These glycosyltransferases included contig01209 (No. 3 in Figure 5B), contig03646 (No. 6 in Figure 5B), contig05219 (No. 11 in Figure 5B), contig09428 (No. 13 in Figure 5B), contig09463 (No. 14 in Figure 5B) and contig09686 (No. 15 in Figure 5B). These contigs were regarded as candidate glycosyltransferases that encode the enzymes responsible for glycyrrhizin biosynthesis and will be the subject of further study. We did not select singletons that were annotated as cytochrome P450s or glycosyltransferases for the organ-specific expression pattern analysis because of the high content of glycyrrhizin in the *Glycyrrhiza *plant (3 - 5% in the root) [30]. On the other hand, 22 ESTs were annotated as CYP88D6 (contig01314, No. 2 in Figure 5A), which is a known cytochrome P450 gene in the glycyrrhizin biosynthetic pathway. The lists of candidate unigenes for cytochrome P450s and glycosyltransferases are found in Additional files 7 and 8, respectively.

## Conclusions

Our study established a high-quality EST database for *G. uralensis *using 454 GS FLX Titanium sequencing technology. With this work, we initiated a large-scale investigation of the transcriptome of *G. uralensis *in terms of functional genomics, molecular biology and biochemistry. A large number of novel candidate genes involved in glycyrrhizin biosynthesis, including cytochrome P450s and glycosyltransferases, were identified in our EST dataset. The information from these ESTs represents a significant contribution toward the exploration of the molecular mechanisms of glycyrrhizin biosynthesis. More importantly, a few candidate genes encoding the enzymes responsible for glycyrrhizin skeleton modifications were obtained by screening functional annotations and by organ-specific expression pattern analyses.

## Methods

### Plant materials

*G. uralensis *material was collected from a five-year-old, field-grown *G. uralensis *plant growing in Ningxia, China. Previous research has shown that wild *G. uralensis *contains much more glycyrrhizin than cultivated plants [41,42]. One possible reason for this difference is that under cultivated plant conditions, *G. uralensis *grows more vigorously and has a more active primary metabolism, while glycyrrhizin accumulation results from secondary metabolism. Wild *G. uralensis *primarily grows in dry areas where lean soil inhibits vegetative growth and thus favors the synthesis and accumulation of glycyrrhizin. Additional studies have shown that the glycyrrhizin content of the *G. uralensis *plant is related to its growth period [42,43]. In contrast to the chemical methods that are mainly used to investigate glycyrrhizin content and accumulation, our transcriptome sequencing method is designed to only reveal genes that are expressed during sampling. Therefore, to investigate the secondary metabolites of glycyrrhizin, plants should be sampled during the glycyrrhizin biosynthetic period. Since this period has not been well studied thus far, we decided to use five-year-old wild *G. uralensis*.

### RNA extraction and cDNA library synthesis

A mixture of approximately 1 g of roots, 1 g of stems and 1 g of leaves was ground to a fine powder under liquid nitrogen. Total RNA purification was performed with the Universal Plant Total RNA Rapid Extraction kit (Bioteke, China). The poly(A) RNA was then separated from the total RNA using the Oligotex^® ^mRNA kit (Qiagen). The quality and purity of the poly(A) RNA was analyzed with 1.0% agarose gels and a GE GeneQuant 100 Spectrophotometer. Ethidium bromide-stained mRNA appeared as a smear that ranged from 500 to 2,000 bp, and the ratio between the absorbance values at 260 and 280 nm was 2.03. Subsequently, 1 μg of mRNA was using the SMART™ PCR cDNA Synthesis kit (Clontech). The cDNA was amplified using the PCR Advantage II polymerase (Clontech) and the following thermal profile: 1 min at 95°C, and then 12 cycles of 95°C for 15 sec, 65°C for 30 sec and 68°C for 6 min. The amplified cDNA product was purified using the PureLink™ PCR Purification kit (Invitrogen) to remove fragments of less than 300 bp. Finally, approximately 5 μg of the resulting cDNA was used to construct a 454 library.

### 454 library preparation and sequencing

Sequencing was based on the 454 GS FLX platform and Titanium reagents. Preparation of the 454 library was performed according to the supplier's instructions. In summary, approximately 5 μg of amplified cDNA was nebulized and selected for length, which ranged from 300 to 800 bp. The FLX specific adapters, Adapter A (GCCTCCCTCGCGCCATCAG) and Adapter B (GCCTTGCCAGCCCGCTCAG), were added to each fragmented cDNA, resulting in Adapter A-DNA fragment-Adapter B constructs. The DNA fragments were then denatured to generate single-stranded DNA, which was then amplified by emulsion PCR for sequencing. The sequencing of the libraries was performed on a 454 GS FLX platform (454 Life Sciences, Roche).

### Sequence assembly

All processing and analyses of the sequencing data was performed with the GS-FLX Software v2.0.01 (454 Life Sciences, Roche). Using a series of normalization, correction and quality-filtering algorithms, the 454 sequencing data were processed to screen and filter for weak signals and low-quality reads, and to trim the read ends for low-quality and 454 adaptor sequences. The resulting 59,219 HQ reads were then submitted to the Short Read Archive at NCBI and assigned the accession number SRX011915. The HQ reads were combined with the 50,666 *G. uralensis *ESTs in GenBank and filtered, clustered and assembled into transcript contigs using GS *De Novo *Assembler Software, which is an application of the GS FLX Software. The filtering step included the masking of SMART PCR primer sequences (Clontech) and the removing of reads that were shorter than 50 bases. The assembly was conducted using the default parameters. Reads that did not fit into a contig were defined as singletons. The resulting singletons and contigs (unigenes) represented the *G. uralensis *candidate gene set.

### Functional annotation and GO classification

The unigenes were annotated by a BLAST (version Blast 2.2.17) [22] search against a series of protein and nucleotide databases, including the curated protein database of Uniprot/SwissProt (released on 06/19/2009) [23], the KEGG GENE database (version KEGG 50) [24], the *Arabidopsis thaliana *proteome databases (version TAIR9) [25] and the NCBI non-redundant protein (Nr) [26] and nucleotide (Nt) [27] databases (released on 06/23/2009). The unigenes were compared against these databases with a significance threshold of e-value ≤ le-5. To maximize computational speed, the search was limited to the first five significant hits for each query. The definition line of the top BLAST hit was used as a description of the putative function of the queried unigene. Customized Perl scripts were used to parse the BLAST outputs.

The Gene Ontology annotations were assigned based on similarity to the *A. thaliana *proteomic sequences (TAIR9) [25,29]. This database was chosen because it has been extensively annotated in GO terms. Each of the unigenes was assigned a GO term based on the top BLAST hit for that query. The transcripts were classified into 45 GO categories under the major categories of Cellular Component, Molecular Function and Biological Process.

### Gene discovery and classification for glycyrrhizin biosynthesis

To evaluate the completeness of our transcriptome library and the effectiveness of our annotation procedure, we searched the annotated sequences for genes involved in the glycyrrhizin metabolic pathway. These simple text searches were based on standard gene names or synonyms.

### Real-time PCR

The mRNA levels of selected *cytochrome P450s *and *glycosyltransferases *genes in different *G. uralensis *organ types were analyzed by RT-PCR. Reverse transcription was performed with DNase I-treated total RNA of *G. uralensis *roots, stems and leaves using the PrimeScript™ 1st Strand cDNA Synthesis Kit (TaKaRa, Dalian, China). The quantitative reaction was performed on an IQ5 Multicolor Real-Time PCR Detection System (Bio-Rad, USA) using SYBR Premix Ex Taq™ (TaKaRa, Dalian, China). PCR amplification was performed under the following conditions: 2 min at 50°C and 30 sec at 95°C, and then 40 cycles of 95°C for 15 sec and 62°C for 1 min. The gene expression of *cytochrome P450s *and *glycosyltransferases *was normalized against an internal reference gene, *glyceraldehyde-3-phosphate dehydrogenase *(*GAPDH*), which was found in our EST library. All primers used in this study are listed in Additional file 5.

## List of abbreviations

cDNA: complementary DNA; EST: expressed sequence tag; bp: base pairs; GuEST: *G. uralensis *ESTs derived from GenBank; BLAST: Basic Local Alignment Search Tool; GO: Gene Ontology; KEGG: Kyoto Encyclopedia of Genes and Genomes; NCBI: National Center for Biotechnology Information; Nr: non-redundant protein data bank; Nt: Entrez nucleotide database; TAIR: The Arabidopsis Information Resource.

## Authors' contributions

YL contributed to the tissue sample collection, RNA extraction, cDNA library construction, bioinformatic analysis and writing of the manuscript. HML helped with the RNA extraction, construction of the cDNA library and writing of the manuscript. CS helped to prepare the first draft of the manuscript and discussed the results. JYS initiated the EST project and helped with the construction of the cDNA library. YZS performed the real-time PCR and the corresponding data analysis. QW aided in the RNA extraction. NW contributed to revisions of the manuscript. HY helped with the RNA extraction and the construction of the cDNA library. AS contributed to the discussion of the gene candidates for the biosynthesis of secondary metabolites and the revisions of the manuscript. This work was conducted in the laboratory of SLC, who initiated the 454 sequencing project and contributed to the evaluation and discussion of the results, as well as to the revisions of the manuscript. All authors contributed to the content of the manuscript, and have read and approved the final version.

## Supplementary Material

Additional file 1 **Percentages of EST having hits in major public databases**. Word document containing the hit numbers and percentages relative to those of the major public databases, including SwissProt, KEGG, TAIR, Nr and Nt. Click here for file

Additional file 2 **The putative glycyrrhizin biosynthetic pathway**. Word document containing the putative glycyrrhizin biosynthetic pathway of *G. uralensis*. Click here for file

Additional file 3 **Gene discovery for glycyrrhizin skeleton synthesis**. Excel document containing the annotations of putative genes corresponding to the glycyrrhizin skeleton synthesis. Click here for file

Additional file 4 **Classification of the candidate P450 genes**. Word document containing the classification of the candidate P450 genes by CYP families. Click here for file

Additional file 5 **Primers used in this study**. Excel document containing the primers used in this study. Click here for file

Additional file 6 **Classification of the candidate glycosyltransferase genes**. Word document containing the classification of the candidate glycosyltransferase genes according to the GO category. Click here for file

Additional file 7 **Cytochrome P450 gene discovery**. Excel document containing the annotations of putative genes annotated as cytochrome P450. Click here for file

Additional file 8 **Glycosyltransferase gene discovery**. Excel document containing the annotations of putative genes annotated as glycosyltransferase. Click here for file
