# Supplementary material for: EST analysis reveals putative genes involved in glycyrrhizin biosynthesis
Source: BMC Genomics. 2010 Apr 28;11:268. doi: 10.1186/1471-2164-11-268 (PMC2886062; doi:10.1186/1471-2164-11-268)
Supplement: Additional file 1 — Percentages of EST having hits in major public databases. Word document containing the hit numbers and percentages relative to those of the major public databases, including SwissProt, KEGG, TAIR, Nr and Nt. [file 1471-2164-11-268-S1.DOC]

## Additional file 1 - Hit percentages against important public databases^a^

|  | **27,229 unigenes** | |
| --- | --- | --- |
| **database** | **annotated (n)** | **percentage (%)** |
| SwissProt | 9,150 | 33.6 |
| KEGG | 14,231 | 52.3 |
| TAIR | 16,226 | 59.6 |
| Nr | 17,520 | 64.3 |
| Nt | 19,138 | 70.3 |
| total | 20,437 | 75.1 |

^a^E value threshold is 1e-5.
