# Supplementary material for: EST analysis reveals putative genes involved in glycyrrhizin biosynthesis
Source: BMC Genomics. 2010 Apr 28;11:268. doi: 10.1186/1471-2164-11-268 (PMC2886062; doi:10.1186/1471-2164-11-268)
Supplement: Additional file 2 — The putative glycyrrhizin biosynthetic pathway. Word document containing the putative glycyrrhizin biosynthetic pathway of G. uralensis. [file 1471-2164-11-268-S2.DOC]

### Additional file 2 –The putative glycyrrhizin biosynthetic pathway of *G. uralensis*

A - The synthesis of the biochemically active isoprene units

B - The synthesis of the glycyrrhizin skeleton

C - glycyrrhizin skeleton modifications

The putative glycyrrhizin biosynthetic pathway in *G. uralensis*. This pathway involves the synthesis of biochemically active isoprene units (A), followed by the synthesis of the triterpene skeleton β-amyrin (B) and then a series of skeleton modifications leading from β-amyrin to glycyrrhizin (C).

The compound names are as follows:

(1) acetyl-CoA, (2) acetoacetyl-CoA, (3) HMG-CoA, (4) mevalonate,

(5) mevalonate-5P, (6) mevalonate-5PP, (7) IPP, (8) DMAPP, (9) pyruvate,

(10) D-glyceraldehyde 3-phosphate, (11) 1-Deoxy-D-xylulose 5-phosphate,

(12) 2-C-methyl-D-erythritol 4-phosphate,

(13) 4-(cytidine 5’-diphospho)-2-C-methyl-D-erythntol

(14) 2-phospho-4-(cytidine 5’-diphospho-2-C-methyl-D-erythritol,

(15) 2-C-methyl-D-erythritol 2,4-cyclodiphosphate,

(16) (E)-4-hydroxy-3-methylbut-2-enyl-diphosphate,

(17) farnesyl-diphosphate, (18) squalene, (19) squalene-2,3-oxide, (20) β-amyrin,

(21) 11α-hydroxy- β-amyrin, (22) 11-oxo-β-amyrin,

(23) 30-hydroxy-11-oxo-β-amyrin, (24) 30-hydroxy-β-amyin,

(25) 11α, 30-dihydroxy-β-amyrin, (26) glycyrrhetinic acid, (27) glycyrrhizin

**References**

1. Seki H, Ohyama K, Sawai S, Mizutani M, Ohnishi T, Sudo H, Akashi T, Aoki T, Saito K, Muranaka T: **Licorice beta-amyrin 11-oxidase, a cytochrome P450 with a key role in the biosynthesis of the triterpene sweetener glycyrrhizin.** *Proc Natl Acad Sci U S A 2008,* **105**(37):14204-14209.
2. Dewick PM: *Medicinal Natural Products: A Biosynthetic Approach*. Wiley, 2009.
