# Supplementary material for: EST analysis reveals putative genes involved in glycyrrhizin biosynthesis
Source: BMC Genomics. 2010 Apr 28;11:268. doi: 10.1186/1471-2164-11-268 (PMC2886062; doi:10.1186/1471-2164-11-268)
Supplement: Additional file 4 — Classification of the candidate P450 genes. Word document containing the classification of the candidate P450 genes by CYP families. [file 1471-2164-11-268-S4.DOC]

### Additional file 4 –Classification of the candidate P450 genes

|  | **number of subfamily** | **number of unigene** | **total EST number** | **number of 454EST** | **number of GuEST** |
| --- | --- | --- | --- | --- | --- |
| CYP4 | 2 | 3 | 3 | 3 | 0 |
| CYP51 | 1 | 1 | 16 | 5 | 11 |
| CYP71 | 3 | 12 | 42 | 33 | 9 |
| CYP72 | 1 | 11 | 78 | 71 | 7 |
| CYP73 | 1 | 2 | 18 | 1 | 17 |
| CYP74 | 1 | 2 | 45 | 0 | 45 |
| CYP75 | 2 | 3 | 10 | 7 | 3 |
| CYP76 | 3 | 8 | 25 | 17 | 8 |
| CYP77 | 1 | 1 | 11 | 2 | 9 |
| CYP78 | 1 | 2 | 3 | 2 | 1 |
| CYP81 | 2 | 9 | 26 | 25 | 1 |
| CYP82 | 1 | 12 | 64 | 37 | 27 |
| CYP83 | 1 | 1 | 17 | 12 | 5 |
| CYP84 | 1 | 3 | 4 | 3 | 1 |
| CYP85 | 1 | 1 | 1 | 1 | 0 |
| CYP86 | 2 | 2 | 6 | 1 | 5 |
| **CYP88** | **1** | **2** | **23** | **20** | **3** |
| CYP89 | 1 | 2 | 9 | 3 | 6 |
| CYP90 | 3 | 7 | 10 | 8 | 2 |
| **CYP93** | **2** | **6** | **30** | **24** | **6** |
| CYP94 | 3 | 5 | 5 | 3 | 2 |
| CYP97 | 3 | 8 | 11 | 11 | 0 |
| CYP98 | 1 | 1 | 4 | 0 | 4 |
| CYP701 | 1 | 2 | 3 | 3 | 0 |
| CYP704 | 1 | 2 | 4 | 1 | 3 |
| CYP705 | 1 | 1 | 1 | 1 | 0 |
| CYP707 | 1 | 6 | 11 | 8 | 3 |
| CYP711 | 1 | 2 | 2 | 1 | 1 |
| CYP716 | 1 | 2 | 6 | 5 | 1 |
| CYP722 | 1 | 1 | 2 | 1 | 1 |
| CYP725 | 1 | 1 | 1 | 0 | 1 |
| CYP750 | 1 | 1 | 6 | 3 | 3 |
| unclassified CYP | 0 | 3 | 3 | 2 | 1 |
| total | 47 | 125 | 500 | 314 | 186 |
