# Supplementary material for: EST analysis reveals putative genes involved in glycyrrhizin biosynthesis
Source: BMC Genomics. 2010 Apr 28;11:268. doi: 10.1186/1471-2164-11-268 (PMC2886062; doi:10.1186/1471-2164-11-268)
Supplement: Additional file 6 — Classification of the candidate glycosyltransferase genes. Word document containing the classification of the candidate glycosyltransferase genes according to the GO category. [file 1471-2164-11-268-S6.DOC]

### Additional file 6 –Classification of the candidate glycosyltransferase genes

| **GO terms for glycosyltransferase** | **Numbers of unigene** | **Numbers of EST** | **Numbers of 454EST** | **Numbers of GuEST** |
| --- | --- | --- | --- | --- |
| 1,3-beta-glucan synthase activity | 6 | 9 | 9 | 0 |
| 1,4-alpha-glucan branching enzyme activity | 3 | 331 | 299 | 32 |
| 4-alpha-glucanotransferase activity | 4 | 32 | 31 | 1 |
| 4-galactosyl-N-acetylglucosaminide 3-alpha-L-fucosyltransferase activity | 2 | 2 | 2 | 0 |
| abscisic acid glucosyltransferase activity | 1 | 4 | 2 | 2 |
| adenine phosphoribosyltransferase activity | 2 | 44 | 9 | 35 |
| alpha,alpha-trehalose-phosphate synthase (UDP-forming) activity | 4 | 13 | 13 | 0 |
| alpha-1,3-mannosylglycoprotein 2-beta-N-acetylglucosaminyltransferase activity | 2 | 5 | 4 | 1 |
| alpha-1,3-mannosyltransferase activity | 1 | 5 | 5 | 0 |
| alpha-1,4-mannosyltransferase activity | 1 | 1 | 0 | 1 |
| amidophosphoribosyltransferase activity | 3 | 4 | 1 | 3 |
| ATP phosphoribosyltransferase activity | 2 | 15 | 12 | 3 |
| cellulose synthase activity | 14 | 39 | 37 | 2 |
| cis-zeatin O-beta-D-glucosyltransferase activity | 1 | 1 | 1 | 0 |
| digalactosyldiacylglycerol synthase activity | 1 | 6 | 0 | 6 |
| dolichyl-diphosphooligosaccharide-protein glycotransferase activity | 3 | 33 | 24 | 9 |
| flavonol 3-O-glucosyltransferase activity | 1 | 4 | 4 | 0 |
| fucosyltransferase activity | 3 | 4 | 4 | 0 |
| galactinol-sucrose galactosyltransferase activity | 1 | 1 | 1 | 0 |
| galactolipid galactosyltransferase activity | 1 | 7 | 4 | 3 |
| **glucuronosyltransferase activity** | **11** | **33** | **17** | **16** |
| mannosyltransferase activity | 3 | 5 | 4 | 1 |
| NAD+ ADP-ribosyltransferase activity | 1 | 4 | 4 | 0 |
| nicotinate phosphoribosyltransferase activity | 1 | 7 | 7 | 0 |
| oligosaccharyl transferase activity | 2 | 12 | 11 | 1 |
| orotate phosphoribosyltransferase activity | 3 | 7 | 2 | 5 |
| phosphorylase activity | 7 | 74 | 66 | 8 |
| protein N-acetylglucosaminyltransferase activity | 2 | 5 | 4 | 1 |
| sinapate 1-glucosyltransferase activity | 2 | 2 | 2 | 0 |
| sucrose synthase activity | 5 | 51 | 18 | 33 |
| sucrose-phosphate synthase activity | 4 | 16 | 12 | 4 |
| transferase activity | 4 | 11 | 1 | 10 |
| transferase activity, transferring glycosyl groups | 114 | 460 | 309 | 151 |
| transferase activity, transferring hexosyl groups | 21 | 35 | 20 | 15 |
| trans-zeatin O-beta-D-glucosyltransferase activity | 1 | 1 | 1 | 0 |
| UDP-galactosyltransferase activity | 2 | 13 | 4 | 9 |
| UDP-glucose:glycoprotein glucosyltransferase activity | 1 | 1 | 1 | 0 |
| **UDP-glucosyltransferase activity** | **27** | **83** | **42** | **41** |
| UDP-glycosyltransferase activity | 45 | 187 | 98 | 89 |
| UDP-sulfoquinovose:DAG sulfoquinovosyltransferase activity | 5 | 23 | 23 | 0 |
| UDP-xylosyltransferase activity | 3 | 3 | 1 | 2 |
| uracil phosphoribosyltransferase activity | 1 | 7 | 3 | 4 |
| xyloglucan 6-xylosyltransferase activity | 4 | 11 | 1 | 10 |
| xyloglucan:xyloglucosyl transferase activity | 15 | 200 | 87 | 113 |
| xylosyltransferase activity | 1 | 1 | 0 | 1 |
| Unclassified | 5 | 5 | 2 | 3 |
